# Alterations of the fecal microbiota in relation to acute COVID-19 infection and recovery

**DOI:** 10.1186/s43556-022-00103-1

**Published:** 2022-11-28

**Authors:** Yue Sandra Yin, Carlos D. Minacapelli, Veenat Parmar, Carolyn C. Catalano, Abhishek Bhurwal, Kapil Gupta, Vinod K. Rustgi, Martin J. Blaser

**Affiliations:** 1 Center for Advanced Biotechnology and Medicine, Rutgers University, Piscataway, NJ USA; 2 Division of Gastroenterology and Hepatology, Rutgers Robert Wood Johnson School of Medicine, New Brunswick, NJ USA; 3 Center for Liver Diseases and Liver Masses, Rutgers Robert Wood Johnson Medical School, New Brunswick, NJ USA

**Keywords:** SARS-CoV-2, Microbiome, Infectious diseases, Clinical infection, Community ecology, Gastrointestinal tract

## Abstract

People with acute COVID-19 due to SARS-CoV-2 infection experience a range of symptoms, but major factors contributing to severe clinical outcomes remain to be understood. Emerging evidence suggests associations between the gut microbiome and the severity and progression of COVID-19. To better understand the host-microbiota interactions in acute COVID-19, we characterized the intestinal microbiome of patients with active SARS-CoV-2 infection in comparison to recovered patients and uninfected healthy controls. We performed 16S rRNA sequencing of stool samples collected between May 2020 and January 2021 from 20 COVID-19-positive patients, 20 COVID-19-recovered subjects and 20 healthy controls. COVID-19-positive patients had altered microbiome community characteristics compared to the recovered and control subjects, as assessed by both α- and β-diversity differences. In COVID-19-positive patients, we observed depletion of Bacteroidaceae, Ruminococcaceae, and Lachnospiraceae, as well as decreased relative abundances of the genera *Faecalibacterium, Adlercreutzia*, and the *Eubacterium brachy* group. The enrichment of Prevotellaceae with COVID-19 infection continued after viral clearance; antibiotic use induced further gut microbiota perturbations in COVID-19-positive patients. In conclusion, we present evidence that acute COVID-19 induces gut microbiota dysbiosis with depletion of particular populations of commensal bacteria, a phenomenon heightened by antibiotic exposure, but the general effects do not persist post-recovery.

**Supplementary Information:**

The online version contains supplementary material available at 10.1186/s43556-022-00103-1.

## Introduction

Since its introduction into human populations in late 2019, severe acute respiratory syndrome coronavirus 2 (SARS-CoV-2) infections have been marked by extreme variability in clinical outcomes, ranging from asymptomatic infection to death [1–3]. Even among those with symptomatic infection, called the coronavirus disease 2019 (COVID-19), clinical severity has been quite variable [4, 5]. Worldwide, most of the infected patients have recovered from the disease, as defined by SARS-CoV-2 viral clearance. However, many have suffered from persistent and sometimes different symptoms after acute COVID-19 [6–8]. In the past 30 months, several factors associated with differences in clinical manifestations and in recovery have been identified including sex, obesity, and presence of comorbidities such as cardiovascular disease and diabetes, but the most important risk factor is advanced age [5, 9–11]. Nevertheless, the major factors leading to severe outcomes only account for a portion of the risk [9, 12].

Several studies have reported the prevalence of gastrointestinal (GI) symptoms at the presentation of COVID-19 and the consistent detection of viral shedding in the stools of patients, suggesting a substantial involvement of the GI tract in acute COVID-19 infection [13, 14]. Therefore, one host factor that could modulate clinical differences is the state of the host microbiota. Humans carry very large and diverse populations of microbes, termed the microbiome, living in the GI tract, skin, and other organs [15, 16]. The largest population is in the colon, and it interacts with human metabolism, immunity, and the central nervous system [17–21]. Despite conserved similarities in its population structure [22], there is extensive inter-personal variation in the types and abundances of the bacterial taxa present [23, 24].

Due to this variation, and the general importance of the gut microbiome in host defenses against infections, there has been interest in the characteristics of the host microbiome as a determinant of the interaction of SARS-CoV-2 with humans [25–34]. We now consider the taxonomic characteristics of the intestinal microbiome, as assessed from fecal samples, in patients with active SARS-CoV-2 infections and those at least 2 weeks after viral clearance in relation to uninfected persons. We find that acute COVID-19 induces gut microbiota dysbiosis with depletion of particular populations of commensal bacteria, but the effect does not persist post-recovery.

## Results

### Subject characteristics of the study cohort

We collected stool samples from 20 subjects each: patients with active COVID-19 infection (Positive), patients recovered from COVID-19 (Recovered), and healthy controls who had not been infected with SARS-CoV-2 (Controls). Demographic and clinical characteristics of these subjects are summarized in Table 1. The COVID-19-positive patients were significantly older than the Controls (*p* = 0.01) and Recovered subjects (*p* = 0.049). All subjects were receiving a regular diet except two COVID-19-positive subjects receiving a low sodium diet. Hypertension was the most common comorbidity among COVID-19-positive patients (50%), followed by obesity (35%) and diabetes mellitus (35%). Comorbidities involving gastrointestinal disorders were observed in 5% of the Controls and 5% of COVID-19-positive patients. During the acute SARS-CoV-2 infection, six (30%) patients experienced gastrointestinal symptoms. Subjects across all study groups reported the use of antibiotics in the prior 6 months, including 10% of the Control, 60% of the Positive, and 20% of the Recovered subjects. Among Positive patients, 50% received at least one form of COVID-19 treatment, excluding dietary supplements (Table 1).

**Table 1:** Demographic and clinical characteristics of the 60 study subjects

Variables	Controls	COVID-19-positive	COVID-19-recovered
**Number, n**	20	20	20
Male, %	60.0	60.0	60.0
Median age, y (IQR)^a^	41 (30–50)	58 (40–67)	46 (32–54)
Race, %			
Asian	40.0		20.0
African American	10.0	15.0	10.0
Hispanic		35.0	20.0
White	50.0	50.0	50.0
Comorbidities, %			
Gastrointestinal disorders	5.0	5.0	
Obesity		35.0	
Diabetes mellitus		35.0	
Gastrointestinal symptoms, %		30.0	5.0
Antibiotic usage, %	10.0	60.0	20.0
Cephalosporin		45.0	
Penicillin		35.0	5.0
Glycopeptide		30.0	
Macrolide	5.0	20.0	10.0
Sulfonamide	5.0	10.0	
Unspecified			5.0
Antiviral therapy, %		25.0	
Tocilizumab		20.0	
Unspecified		5.0	
Other COVID-19 treatment, %			
Hydroxychloroquine		30.0	
Dietary supplements		30.0	20.0
Antifungal therapy		10.0	
Corticosteroid		20.0	

^a^Age comparisons were performed using one-way ANOVA: *p* = 0.01 between healthy controls and COVID-19-positive; *p* = 0.049 between COVID-19-positive and COVID-19- recovered; *p* = 0.52 between healthy controls and COVID-19-recovered

### COVID-19 altered gut microbiome community characteristics

**Fig. 1:**
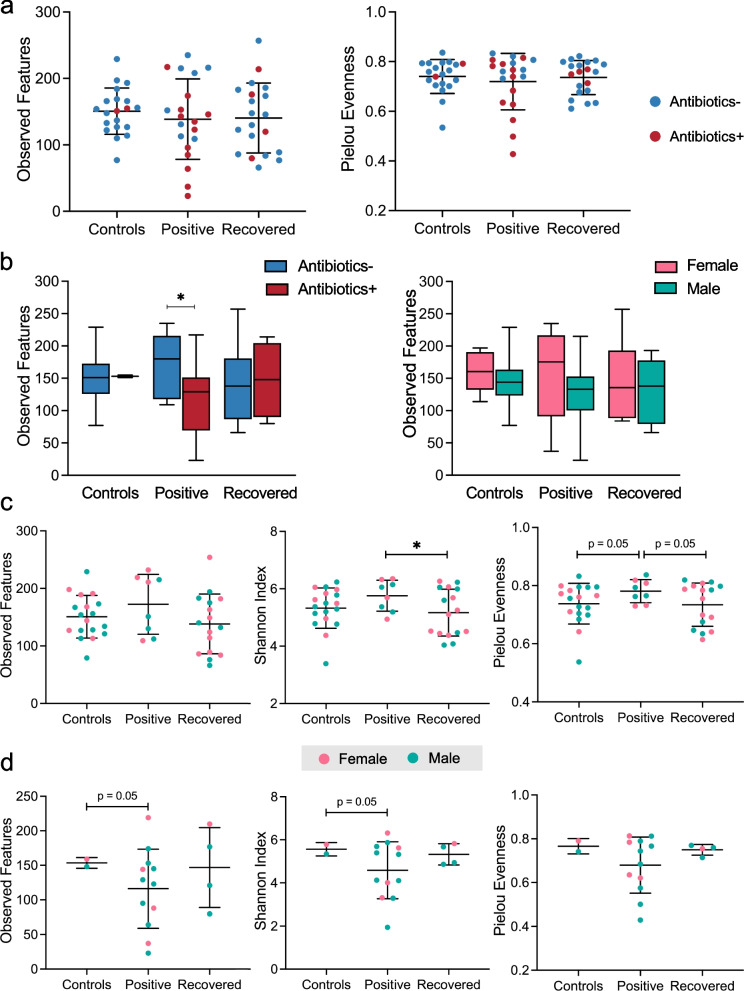
Gut microbial alpha-diversity in three groups of study subjects, based on 16S rRNA sequences in fecal samples. **a** Species richness and evenness of fecal samples from Controls (n = 20), COVID-19-positive patients (n = 20), and COVID-19-recovered patients (n = 20) were measured in terms of observed OTUs and Pielou evenness, respectively. Differences between groups were not significant using Welch’s t-test. **b** Species richness in subjects with or without antibiotic use, and in relation to sex, was estimated by observed OTUs. (Table 1 indicates the number of subjects in each group). *p < 0.05 by Welch’s t-test. **c, d** Alpha-diversity in subjects without (**c**) or with (**d**) antibiotic use was compared between groups using the metrics of observed OTUs, Shannon index, and Pielou evenness. *p < 0.05 by Welch’s t-test

To determine the effects of COVID-19 on the gut microbiota, we examined the abundances of 16S rRNA genes in stool samples. The sequencing generated a mean of 26,775 demultiplexed and denoised operational taxonomic units (OTUs) per sample (Supplementary Fig. 1). The rarefaction curves reached asymptotes, indicating that an even sampling depth of 10,000 reads/sample was sufficient for diversity analyses (Supplementary Fig. 2a). Significant differences in species richness were observed between COVID-19-positive patients and the Controls, as well as between the Controls and Recovered subjects at all sampling depth above 1000 (Supplementary Fig. 2a). No significant difference was detected between COVID-19-positive patients and the Recovered group (Supplementary Fig. 2a). Analysis of α-diversity by observed features and Pielou’s evenness using the rarefied data did not show any significant differences between study groups (Fig. 1a). Antibiotic use was associated with markedly reduced species richness in COVID-19-positive patients while the Controls and Recovered patients had no significant differences (Fig. 1b). There were no significant differences in α-diversity in relation to subject sex in any group regardless of antibiotic exposure (Fig. 1b and Supplementary Fig. 2b). Among all subjects without antibiotic use, COVID-19-positive patients had the highest α-diversity compared to the Control and Recovered subjects. Differences were particularly significant between the Positive and Recovered patients, as determined by the Shannon’s index distance metric (Fig. 1c). The use of antibiotics reversed the effect, contributing to reduced species richness and evenness in COVID-19-positive patients compared to the Controls and Recovered patients (Fig. 1d); however, there were only few cases of antibiotic use among the Controls and Recovered subjects.

**Fig. 2:**
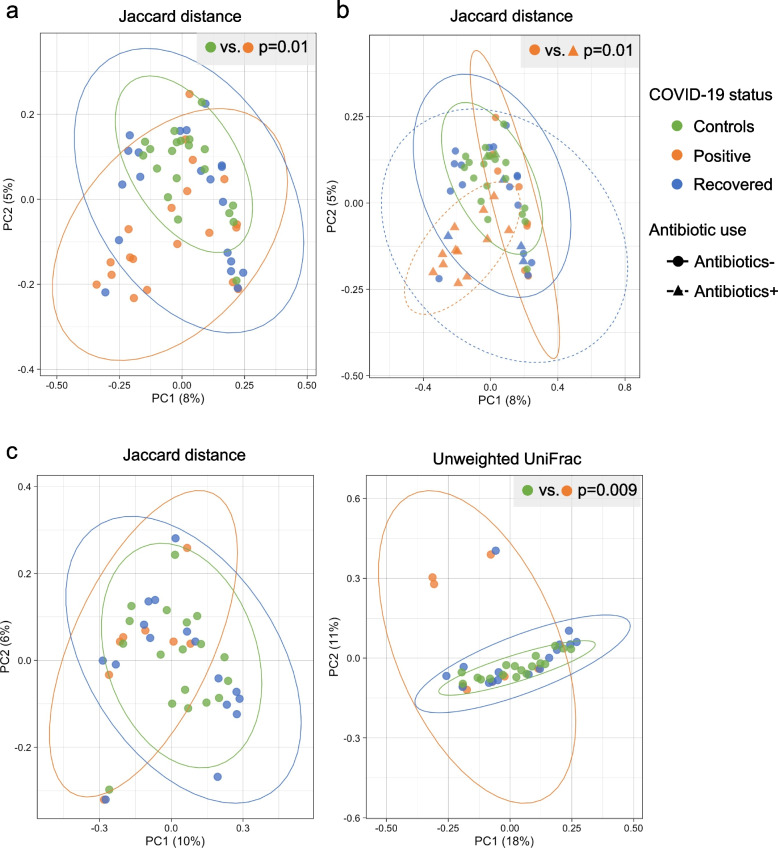
Gut microbial beta-diversity in three groups of study subjects, based on 16S rRNA sequences in fecal samples. In all panels, the ellipses represent 95% confidence intervals, and *p*-values were obtained by PERMANOVA. **a, b** Principal coordinates analysis (PCoA) was used for visualization of Jaccard dissimilarity of gut microbial communities between the Controls (n = 20), COVID-19-positive (n = 20), and COVID-19-recovered patients (n = 20). **b** Groups of subjects with or without antibiotic use were compared. **c** PCoA based on Jaccard distance and unweighted UniFrac distance are shown for subjects in the absence of recent antibiotic exposure. Table 1 indicates the number of subjects in each group

Community structure (β-diversity) substantially overlapped between Recovered subjects and Controls, but the Controls differed significantly with COVID-19-positive patients (Fig. 2a). Principal coordinate analysis (PCoA) revealed that the dissimilarity of microbial communities observed in the COVID-19-positive patients was primarily driven by antibiotic use (Fig. 2b). Using the unweighted UniFrac analysis of β-diversity for comparing all of the subjects who did not have recent antibiotic exposure showed significant dissimilarity between COVID-19-positive patients and the other two groups (Fig. 2c). In total, these results provide evidence that gut microbiota dysbiosis was present in COVID-19 patients, but the effect was largely related to the use of antibiotics and did not persist post-COVID-19 recovery.

### COVID-19 altered gut microbial taxon abundances

**Fig. 3:**
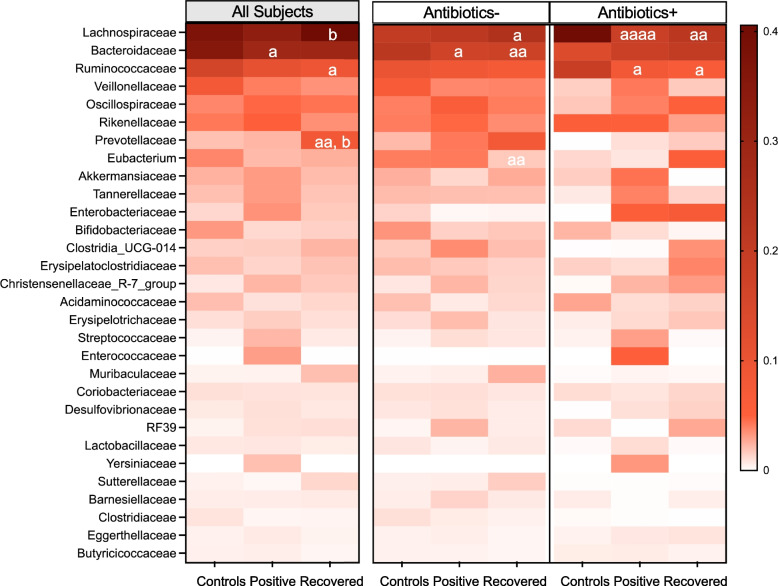
Heatmap of gut microbial compositions in the three groups of study subjects that varied in COVID-19 infection status. The relative abundances of the 30 most abundant bacterial taxa at the family level were compared between the Controls (n = 20), COVID-19-positive (n = 20), and COVID-19-recovered patients (n = 20) (left panel). Additional comparisons were performed in subjects without (center panel) or with recent antibiotic use (right panel). Table 1 indicates the number of subjects in each group. Left panel: statistical significance was assessed for comparisons to all Controls (**a**) or to all COVID-19-positive patients (**b**). For particular taxa: ^a^*p* < 0.05, ^b^*p* < 0.05, and ^aa^*p* < 0.01, by two-way ANOVA. Right panel: *p*-values are shown for comparisons to the Controls (**a**) only between subjects of the same antibiotic use status. Bacterial communities in the COVID-19-positive and COVID-19-recovered patients did not differ substantially, as determined by two-way ANOVA. For particular taxa: ^a^*p* < 0.05, ^aa^*p* < 0.01, and ^aaaa^*p* < 0.0001, by two-way ANOVA

From the 60 stool samples obtained, we identified a total of 122 individual taxa at the family level. Among the 30 most abundant family-level bacterial taxa, Bacteroidaceae and Ruminococcaceae were significantly underrepresented in COVID-19-positive or Recovered patients compared to the Controls, regardless of antibiotic use (Fig. 3). Comparing all subjects, COVID-19 reduced the relative abundance of Lachnospiraceae which was restored to Control levels in the Recovered patients (Fig. 3). In contrast, Prevotellaceae increased in abundance with acute COVID-19 infection which reached the highest levels post-recovery, a trend particularly apparent in subjects without antibiotic use (Fig. 3). COVID-19-recovered patients varied substantially in Eubacterium relative abundance compared to both the Controls and COVID-19-positive patients; however, the effects were opposite in subjects with or without antibiotic use (Fig. 3). No other changes were statistically significant.

**Fig. 4:**
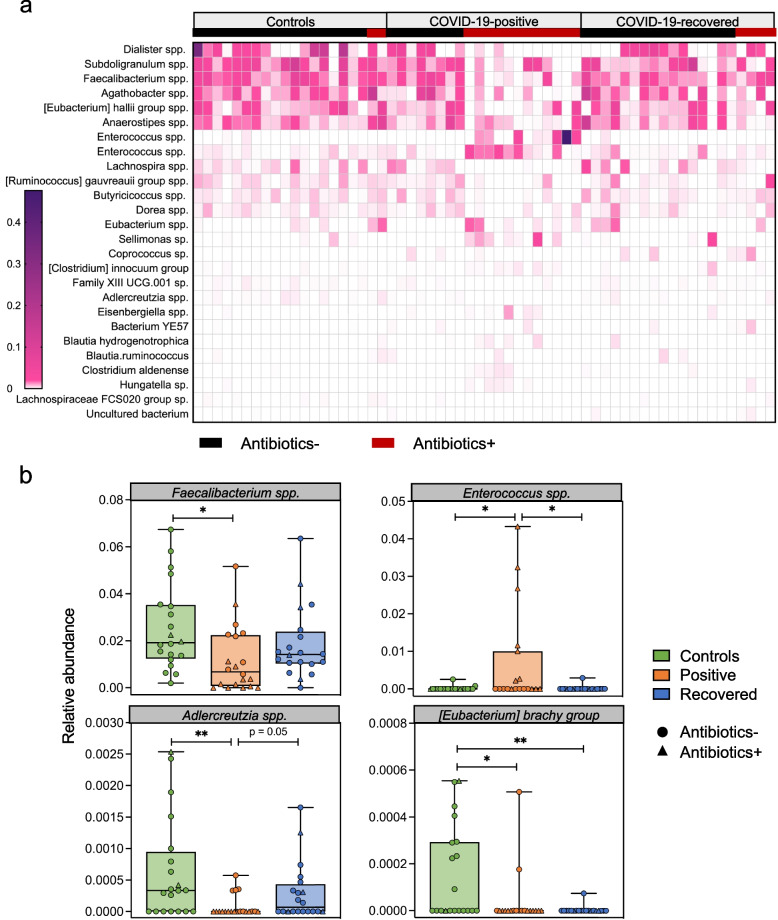
Bacterial species that significantly differ in abundance in groups differing in COVID-19 infection status or antibiotic use status. **a** Heatmap of bacterial species found to significantly differ in abundance between groups differing in COVID-19 infection status or antibiotic use status is shown for the Controls, COVID-19-positive, and COVID-19-recovered patients. Subjects with (red) or without (black) recent antibiotic exposure is indicated in the top line. Table 1 indicates the number of subjects in each group. Differentially abundant species were identified by MaAsLin2 and ordered according to mean relative abundance. **b** Four bacterial species were determined by MaAsLin2 to be significantly differential between the Controls, COVID-19-positive, and COVID-19-recovered patients. Additional statistical comparisons were performed concerning the relative abundance of these species: *p < 0.05 and **p < 0.01 by Welch’s t-test

A total of 26 bacterial species were significantly differential between groups differing in COVID-19 infection status or antibiotic use by MaAsLin 2 (Fig. 4a). In the Controls and Recovered groups, *Dialister, Subdoligranulum, Faecalibacterium, Agathobacter,* and the *Eubacterium hallii* group were highly abundant in most subjects regardless of antibiotic exposure. Lower levels in COVID-19-positive patients were at least in part associated with antibiotic exposure (Fig. 4a). Considering all subjects regardless of antibiotic exposure, four bacterial species (*Faecalibacterium*, *Enterococcus*, *Adlercreutzia*, and the *Eubacterium brachy* group) were significantly differential between groups (Fig. 4b). *Faecalibacterium* and *Adlercreutzia* were significantly reduced in COVID-19 patients while the Controls and Recovered subjects had comparable enrichment (Fig. 4b). The *Eubacterium brachy* group was markedly reduced with COVID-19 infection and remained low post-recovery (Fig. 4b). Taken together, these results provide evidence that COVID-19 induced specific taxonomic changes, which were worse with antibiotic use and which could persist post-recovery.

### Antibiotics further perturbed the gut microbiota in COVID-19 patients

**Fig. 5:**
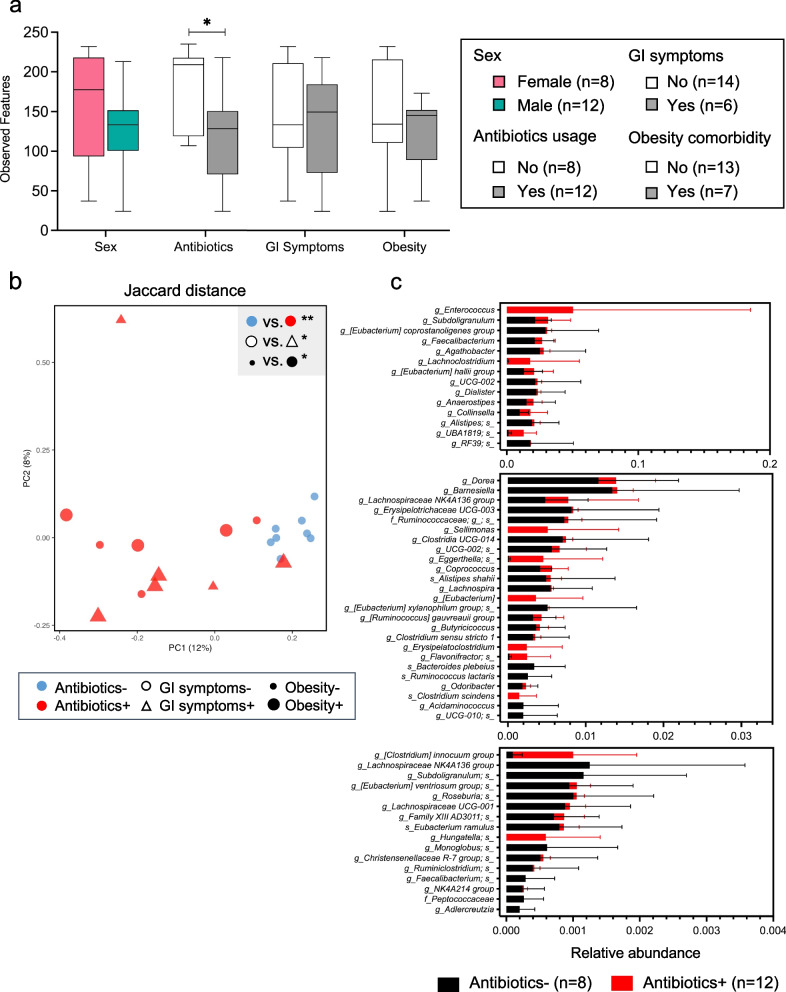
Gut microbial diversity and compositions in COVID-19-positive patients at sequence depth of 10,000. **a** Species richness was estimated by observed OTUs and evaluated based on subject sex, antibiotic use status, and the presence of GI symptoms, or of obesity comorbidity. Statistical significance was determined for comparisons between patients with or without antibiotic use by unpaired Student’s t-test: *p < 0.05. **b** Principal coordinates analysis (PCoA) was used for visualization of Jaccard dissimilarity of gut microbial communities for patients with or without antibiotic use, with or without GI symptoms, or with or without the comorbidity of obesity. The key in Fig. 5a indicates the number of subjects in each group. *p < 0.05 and **p < 0.01 by PERMANOVA. **c** Significantly differential bacterial species in the 20 COVID-19-positive patients, organized according to antibiotic use, were identified by MaAsLin2 and stratified into groups of high- (top panel), medium- (middle panel), and low- (bottom panel) relative abundances

We also evaluated the associations with gut microbial community diversity and composition of host factors (sex, antibiotic exposure, gastrointestinal (GI) symptoms, and obesity comorbidity). Among the COVID-19-positive patients, only antibiotic exposure was significantly associated with altered species richness (α-diversity) (Fig. 5a). COVID-19-positive patients showed distinct β-diversity clustering according to antibiotic use; however, their gut microbial community structure also varied with respect to GI symptoms and obesity (Fig. 5b). We identified 55 bacterial species that differed significantly between COVID-19-positive patients with or without recent antibiotic exposure (Fig. 5c). In total, these data collectively provide evidence that non-viral factors, especially antibiotic use, contributed to the perturbation of the gut microbiota observed in the COVID-19-positive patients.

## Discussion

Emerging evidence suggests that COVID-19 infection is associated with dysbiosis of the gut microbiota [25–34]; however, many of these observational studies were conducted on hospitalized adults from a single country, China, early in the COVID-19 pandemic [25–33, 35]. Longitudinal studies from multiple countries and ethnicities are needed to understand the clinical impact of the acute infection on the microbiome, and vice versa. In this cross-sectional study, we evaluated gut microbial characteristics in healthy controls, hospitalized and non-hospitalized COVID-19-positive patients, as well as recovered patients from different ethnic groups. We now provide evidence that acute COVID-19 infection is associated with gut microbiota perturbations which did not persist post-recovery, and which were highly associated with antibiotic exposure. However, some effects on specific taxa observed in COVID-19 patients continued into recovery despite viral clearance.

Using two α-diversity metrics, we did not detect any significant differences in species richness and evenness between our study groups, consistent with other studies, including those that stratified COVID-19-positive patients according to disease severity [27–29, 31, 34]. Although significantly reduced gut microbiome α-diversity in COVID-19-positive patients compared to healthy controls has been reported [25, 33], some studies did not control for antibiotic use [25]. Our observation of increased species richness and evenness in COVID-19-positive patients compared to healthy controls, in the absence of antibiotic exposure, differed from other observations [29, 31, 33]. These differences may be explained by the demographic and associated lifestyle variation in study cohorts. Our study group was more ethnically diverse compared to prior studies exclusively focused on Chinse subjects [29, 31, 33]. Substantial gut microbiome variation observed between ethnically diverse subjects [36], are partially driven by lifestyle and dietary differences [37]. As such, our current study complements and extends the literature concerning the gut microbiome in COVID-19 by including a study population of diverse characteristics.

We observed distinct gut microbiota composition in COVID-19-positive patients compared to healthy controls regardless of antibiotic exposure. This β-diversity dissimilarity also has been previously observed, providing evidence that acute COVID-19 infection could induce gut microbiota dysbiosis [25, 29, 30, 32–34]. Zhang *et al.* provided further evidence that the dysbiosis might be associated with COVID-19 disease severity as significant β-diversity differences were only observed between COVID-19-patients with severe/critical illness and control subjects without COVID-19 [32]. Our analyses of α- and β-diversity demonstrated that COVID-19-recovered patients and healthy controls had comparable gut microbiomes, which were distinct from those of COVID-19-positive patients. Similarly, in a North American cohort, the microbiota of recovered patients was comparable with control subjects [34]. In contrast, in several Chinese cohorts, the COVID-19-induced gut microbiota dysbiosis persisted after SARS-CoV-2 viral clearance [26, 29]. In addition to demographic differences, these studies exclusively involved hospitalized COVID-19-positive patients who were followed into recovery [26, 29]. Intensive Care Unit (ICU) stays during hospitalization also are known to contribute to gut dysbiosis [38, 39]. Not following the COVID-19-positive patients into recovery in our study, and using unrelated recovered subjects could introduce added inter-personal variation. As such, expanded cohorts that control for these important circumstances are needed to understand the longitudinal changes of the gut microbiome in COVID-19 patients.

In COVID-19-positive patients, we observed depletion of Bacteroidaceae, Ruminococcaceae, and Lachnospiraceae, which are major families present in the healthy adult gut microbiota [17, 40]. Decreased abundances of these commensals have been detected in patients with immune-mediated inflammatory diseases, including inflammatory bowel disease [17, 41], multiple sclerosis [42], and ankylosing spondylitis [43]. Loss of the commensal gut microbiota may attenuate defenses against respiratory pathogens [44], suggesting an interaction with SARS-CoV-2 infection. The genera *Faecalibacterium, Adlercreutzia*, and the *Eubacterium brachy* group were significantly less abundant in COVID-19-positive patients compared to healthy controls, consistent with prior reports of COVID-19-associated depletion of short-chain fatty acids (SCFA)-producing bacteria, including Ruminococcaceae (includes *Faecalibacterium*) and Lachnospiraceae [28, 29, 32, 45, 46]. Since SCFAs have strong anti-inflammatory activities [47], modulating interferon responses to viral infection [48], reduced numbers of SCFA-producing bacteria could interfere with host immune responses against SARS-CoV-2 infection. Recent studies reported enriched levels of *Faecalibacterium* [49], *Ruminococcus* (two representative genera of Ruminococcaceae), and SCFAs [50] in COVID-19 patients who had less severe clinical outcomes, further suggesting an immune-metabolism-microbiome interactive effect on COVID-19 disease severity. In our study, we observed that Ruminococcaceae continued to be depressed in Recovered patients; at the genus level, the *Faecalibacterium* relative abundance also did not recover to that of healthy controls. These findings were consistent with the taxonomic changes observed in a prior study of patients who had severe COVID-19 6 months after recovery [49]. Prevotellaceae and the *Eubacterium brachy* group species were enriched and depleted, respectively, in patients with acute COVID-19, and after viral clearance, their relative abundances continued to change. Prevotellaceae family members can induce periodontitis [51] which has been associated with increased risk of COVID-19 complications [52]. Prolonged changes in Prevotellaceae relative abundance may explain persistent oral manifestations observed in people with long COVID, or post-COVID conditions [53]. Taken together, these findings suggest impact of COVID-19-associated depletion of particular commensal populations on disease course and long-term recovery.

Our study has limitations, including enrolling subjects with varying degrees of exposure to antibiotics – major disruptors of the gut microbiota [15, 54]. As such, this differential exposure confounds comparisons between study groups and identifying true COVID-19 associated microbial signatures; however, it provided an opportunity to study the gut microbiome in COVID-19 patients with and without antibiotics. Antibiotics treatment in COVID-19 patients further shifted the gut microbiome away from the healthy controls, with depletion of many commensals, consistent with prior studies [26, 28, 29]. In prior studies, antibiotic use did not improve COVID-19 outcomes [28], nor did microbiome effects persist beyond 6 months [29]. Altogether these studies further indicate the importance of developing guidelines on antibiotic use in COVID-19 treatment. Age-related loss of gut microbiota diversity also can occur [55]; however, significant changes have mostly been detected at the extremes of age (most notably in centenarians) and in persons residing in long-term care facilities [56–58]. Therefore, although the subjects in this study were not age-matched, we expect little impact of age on the core microbiota between study groups. Studies have shown that COVID-19-associated social and behavior changes, such as lockdown, social distancing [59], and increased use of disinfectants [60] may also impact the gut microbiome. Despite our efforts to minimize such impact and recruit patients who had adopted similar measures by conducting the study at a single site, we recognize the importance of these changes, which could interfere with identifying the direct effects of SARS-CoV-2 on gut dysbiosis. Similarly, our study did not control for past infections with SARS-CoV-2 that were asymptomatic and unknown to the study participants, which also might have perturbed the gut microbiome, especially in healthy controls. Although our findings suggest that the COVID-19 effects on the microbiota do not persist post-recovery, further studies that incorporate antibody test results and viral load will enhance understanding of the impact on the gut microbiome of COVID-19, including disease severity and clinical manifestations.

In conclusion, we present evidence that acute COVID-19 infection can induce gut microbiota dysbiosis with depletion of commensal bacteria, a phenomenon enhanced by antibiotic exposure. Further investigation of patients across the severity gradient in expanded longitudinal cohorts will enhance understanding of the role of the gut microbiome in COVID-19 disease progression and recovery. These findings may help identify microbial targets and probiotic supplements for improving COVID-19 treatment.

## Materials and methods

### Subject recruitment and sample collection

From May 14 2020 to January 28 2021, a total of 60 subjects were recruited into this study at the Robert Wood Johnson University Hospital (RWJUH) in New Brunswick NJ. The study group consisted of 20 COVID-19 (SARS-CoV-2-positive) patients, 20 healthy donors (Controls), and 20 COVID-19-recovered subjects (Recovered). COVID-19-positive patients were recruited from the pool of patients hospitalized at RWJUH, who had diagnosis of SARS-CoV-2 infection, which was confirmed by reverse transcription polymerase chain reaction (RT-PCR) analysis of saliva and/or nasal swabs. Similarly, COVID-19-recovered subjects were selected from the hospital outpatient department and were defined as being > 14 days after SARS-CoV-2 viral clearance (based on a single negative RT-PCR test). Healthy control subjects also were selected among patients and staff at the hospital. Specimens were collected from COVID-19-positive patients within 3 days of onset of illness. Specimens from the Controls and Recovered subjects were collected within 1 day after undergoing the RT-PCR test that showed negativity. Written informed consent was obtained from all subjects before participating in the study.

All of the medical records of the patients were reviewed to collect demographic characteristics, comorbidities, medical history, COVID-19 duration, and treatment. A questionnaire was used at the time of specimen collection to obtain diet type, antibiotics used in the 6 months prior to study entry, and prior and existing treatment for COVID-19. The use of antibiotics also was recorded during hospitalization. Data pertinent to COVID-19 treatment was collected or corroborated during chart review, which included the use of hydroxychloroquine, azithromycin, systemic anticoagulation, corticosteroids, convalescent plasma, and remdesivir. Gastrointestinal (GI) symptoms were recorded as the presence of diarrhea, nausea, and/or vomiting (at least one episode of a self-reported symptom per day prior to testing). Following the Rutgers COVID-19 and laboratory biosafety protocols, stool samples were collected from the recruited subjects into sterile containers using a Protocult collection device (ABC Medical Enterprises Inc., Rochester MN). All samples were de-identified and stored at − 80 °C until analysis.

### DNA extraction and microbiome sequencing

Stool samples were first heated at 60 °C for 60 minutes to inactivate any potentially live viruses. Total DNA was extracted from stool aliquots using the DNeasy PowerSoil HTP 96 Kit (QIAGEN, Valencia CA) following the manufacturer’s instructions. The V4 region of the bacterial 16S rRNA gene was PCR-amplified in triplicate using barcoded fusion primers 515F/806R. The DNA concentration of each amplicon was quantified using the Quant-iT PicoGreen dsDNA Assay Kit (Invitrogen) and the SpectraMax iD3 microplate reader (Molecular Devices, San Jose CA). Samples were pooled and purified with the QIAquick PCR Purification Kit (QIAGEN), and the pooled samples were quantified using the Quant-iT dsDNA Assay Kit, high sensitivity (Invitrogen) on a Qubit 2.0 Fluorometer (Life Technologies, Carlsbad CA) and then combined at equimolar concentrations to form the sequencing library. Paired-end sequencing (2x150bp) of the constructed library was subsequently performed on the Illumina MiSeq platform (Illumina, San Diego CA) at Azenta Life Sciences (South Plainfield NJ). The 16S rRNA sequence data generated for this study have been deposited in QIITA open-source microbiome database (https://qiita.ucsd.edu) under accession number: 14812.

### Bioinformatics analysis of microbiome sequences

Raw paired-end reads with perfect matching of bases between forward and reverse sequences were retained, demultiplexed, filtered, and analyzed using the QIIME 2 v2021.2 pipeline as described [61]. Briefly, the identification of OTUs was performed using the DADA2 plugin with quality filtering to trim the first six bases of each sequence with quality score < 35. Taxonomy was assigned to sequences using the Naïve Bayes classifier compared against a SILVA (138 release) 99% similarity OTUs reference database trained on the 515F/806R region of the 16S rRNA gene [62, 63].

An even sampling depth of 10,000 sequences per sample was used for assessing microbial α- and β-diversity differences. To evaluate species richness and evenness, α-diversity was computed by two commonly used metrics: observed features (or OTUs) and Pielou’s evenness, respectively. In addition, the Shannon’s index was used to estimate both richness and evenness in a single equation. To assess the overall microbial community variation between samples, unweighted UniFrac distance and Jaccard distance matrices were used for β-diversity analysis. The multivariable association analysis with linear models (MaAsLin 2) was implemented in R to detect significant differences in relative abundances of microbial taxa between study groups [64].

### Statistical analysis

Measurements of microbial α-diversity and the relative abundance of bacterial communities and species were analyzed using the GraphPad Prism 9 software and displayed as mean ± standard deviation in scatter plots, box-and-whiskers ± min-to-max, or heatmaps. Comparisons between study groups were assessed by Welch’s t-test or two-way ANOVA to account for additional variables. Statistical significance of inter- and intra-group β-diversity was determined by a permutational multivariate analysis of variance (PERMANOVA). Principal coordinate analysis (PCoA) was applied to create ordinations and to visualize the diversity between samples. A Benjamini-Hochberg-corrected q value < 0.25 was used by MaAsLin2 to determine statistical significance. A *p* value < 0.05 was considered statistically significant for all other statistical analyses throughout this study.

## Supplementary Information

**Additional file 1 Supplementary Fig. 1.** Summary statistics of 16S rRNA gene sequencing depth. The histogram indicates counts of OTUs (frequency) per sample and the number of samples at each depth. Together with the summary statistics, the data indicate 16S rRNA sequencing depth for 60 fecal samples after sequence quality control and feature table construction using DADA2

**Additional file 2 Supplementary Fig. 2.** Gut microbial diversity in three groups of study subjects. **a** Rarefaction analysis of the microbial alpha-diversity of the Controls (n = 20), COVID-19-positive (n = 20), and COVID-19-recovered patients (n = 20) at multiple sampling depths. Alpha-diversity was measured by observed OTUs. One-way ANOVA corrected for multiple comparisons was used to determine statistical significance. **b** Observed OTUs (species richness) at sequence depth of 10,000 in female and male subjects without antibiotic use. Table 1 indicates the number of subjects in each group. No significant differences between the sexes were found, based on Welch’s t-test

## Data Availability

The 16S rRNA sequence data generated for this study have been deposited in QIITA open-source microbiome database (https://qiita.ucsd.edu) under accession number: 14812.

## Abbreviations

SARS-CoV-2: Severe acute respiratory syndrome coronavirus 2
COVID-19: Coronavirus disease 2019
rRNA: Ribosomal RNA
OTU: Operational taxonomic unit
RT-PCR: Reverse transcription–polymerase chain reaction
SCFA: Short-chain fatty acid
