# Supplementary material for: Alterations of the fecal microbiota in relation to acute COVID-19 infection and recovery
Source: Mol Biomed. 2022 Nov 28;3:36. doi: 10.1186/s43556-022-00103-1 (PMC9702442; doi:10.1186/s43556-022-00103-1)
Supplement: Supplementary file 1 — Additional file 1 Supplementary Fig. 1. Summary statistics of 16S rRNA gene sequencing depth. The histogram indicates counts of OTUs (frequency) per sample and the number of samples at each depth. Together with the summary statistics, the data indicate 16S rRNA sequencing depth for 60 fecal samples after sequence quality control and feature table construction using DADA2 [file 43556_2022_103_MOESM1_ESM.pdf]

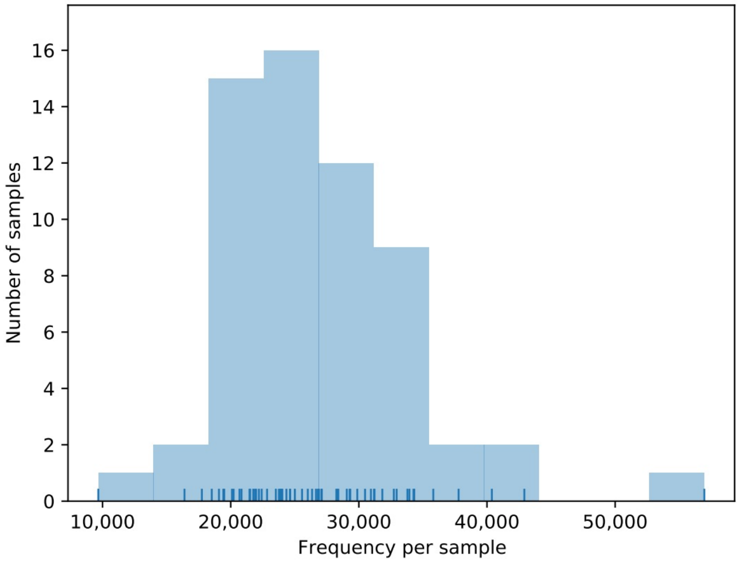

Number of samples	60
Number of unique OTU	2,218
Total frequency	1,606,526
Mean frequency	26,775
Median frequency	25,792
Maximum frequency	56,960
Minimum frequency	9,668
Interquartile range	8,637
