# Supplementary figures and images for: Alterations of the fecal microbiota in relation to acute COVID-19 infection and recovery
Source: Mol Biomed. 2022 Nov 28;3:36. doi: 10.1186/s43556-022-00103-1 (PMC9702442; doi:10.1186/s43556-022-00103-1)

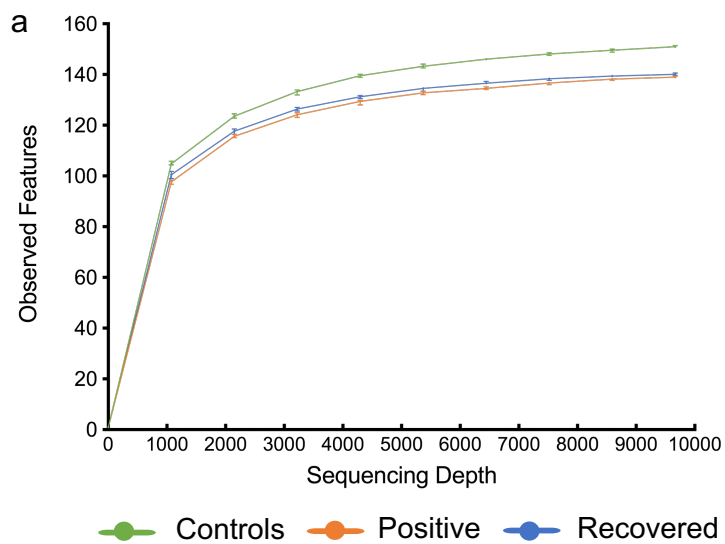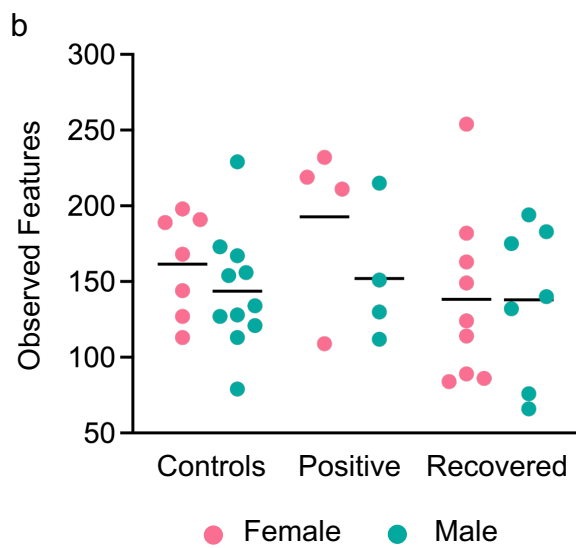

Supplement: Supplementary file 2 — Additional file 2 Supplementary Fig. 2. Gut microbial diversity in three groups of study subjects. a Rarefaction analysis of the microbial alpha-diversity of the Controls (n = 20), COVID-19-positive (n = 20), and COVID-19-recovered patients (n = 20) at multiple sampling depths. Alpha-diversity was measured by observed OTUs. One-way ANOVA corrected for multiple comparisons was used to determine statistical significance. b Observed OTUs (species richness) at sequence depth of 10,000 in female and male subjects without antibiotic use. Table 1 indicates the number of subjects in each group. No significant differences between the sexes were found, based on Welch’s t-test [file 43556_2022_103_MOESM2_ESM.pdf]
